# BRAF in Melanoma: Pathogenesis, Diagnosis, Inhibition, and Resistance

**DOI:** 10.1155/2011/423239

**Published:** 2011-11-17

**Authors:** Ryan J. Sullivan, Keith T. Flaherty

**Affiliations:** ^1^Division of Hematology/Oncology, Beth Israel Deaconess Medical Center, Harvard Medical School, Boston, MA 02114, USA; ^2^Massachusetts General Hospital Cancer Center, Harvard Medical School, Boston, MA 02114, USA

## Abstract

Since the initial discovery that a subset of patients with cutaneous melanoma harbor BRAF mutations, substantial research has been focused on determining the pathologic consequences of BRAF mutations, optimizing diagnostic techniques to identify these mutations, and developing therapeutic interventions to inhibit the function of this target in mutation-bearing tumors. Recently, advances have been made which are revolutionizing the standard of care for patients with BRAF mutant melanoma. This paper provides an overview on the pathogenic ramifications of mutant BRAF signaling, the latest molecular testing methods to detect BRAF mutations, and the most recent clinical data of BRAF pathway inhibitors in patients with melanoma and BRAF mutations. Finally, emerging mechanisms of resistance to BRAF inhibitors and ways of overcoming this resistance are discussed.

## 1. Introduction

Melanoma is currently the 5th and 7th most common cancer in American men and women, respectively [1]. In addition, the incidence of melanoma has risen dramatically over the past 60 years, increasing faster than all other solid tumors [2]. Although early-stage patients can be treated successfully with surgical resection in the majority of patients, many will develop disseminated disease. The prognosis for patients with distant metastases from melanoma is dismal, and despite standard treatment, greater than 95% of patients with stage IV melanoma will die within five years and most patients succumb within one year.

More recently, preclinical discoveries have led to significant advances in the understanding of the key molecular signaling events underlying the pathogenesis of melanoma. Most notably, a high percentage of tumors of melanocytic origin have been shown to harbor activating mutations of BRAF, which lead to its constitutive activity. Approximately 70–80% of acquired melanocytic nevi and 40–60% of malignant melanoma contain a BRAF mutation, the vast majority of which result in a single amino acid change at codon 600 (BRAF^V600E^) [3, 4]. The resultant unopposed, constitutive activation of extracellular signal-regulated kinase (ERK) leads to the promotion of cellular growth and opposition of apoptosis and, ultimately, transformation into melanoma [5]. This enhanced signaling, however, also renders mutated cells susceptible to the use of small molecule inhibitors which target various BRAF pathway mediators [5–7].

## 2. RAF Signaling and Pathogenesis of Melanoma

The interaction between a growth factor receptor and its ligand typically induces a series of events, which promote cellular growth and survival. The RAS family members are GTPases which act as critical mediators in the transduction of such signals. Though RAS plays an important role in the homeostasis of normal cell turnover, death, and survival, activating mutations in RAS family members (HRAS, KRAS, and NRAS) have been identified and associated with various human malignancies [8]. In melanoma, NRAS mutations have been identified in 10–25% of tumor samples and are thought to be an important driver of oncogenesis in these patients [9–12]. Oncogenesis is mediated through the upregulation of several downstream signaling mechanisms, most notably the mitogen-activated protein kinase (MAPK) and the phophatidy-inositol-3-kinase (PI3K) pathways [13]. 

Activated RAS triggers MAPK pathway activation through interactions with the RAF oncoproteins (BRAF and CRAF) leading to the initiation of a progrowth signaling cascade [14]. It is unclear whether it is BRAF or CRAF that transmits signal from mutated NRAS to MEK, but the preponderance of evidence suggests that CRAF is the primary mediator [15]. RAF interacts with MAPK/ERK kinase (MEK) thereby initiating MEK phosphorylation which in turn leads to an activating phosphorylation of ERK [14]. The activation of ERK leads to a progrowth and transforming signal, which appears to be critical to the pathogenesis of many malignancies. This pathway can be initiated by either RAF isoform, BRAF, or CRAF, though CRAF also has pro-survival effects, in part through the upregulation of the anti-apoptotic proteins, nuclear factor kappa B (NF-*κ*B), and B-cell leukemia 2 (BCL-2) [14]. Interestingly, unlike CRAF, activated BRAF has no other known substrates. Thus, BRAF mutant melanomas signal exclusively through MEK and subsequently ERK leading to oncogenesis. This characteristic renders these tumors exquisitely sensitive to potent inhibitors of the MAPK pathway.

## 3. Diagnostics/Detection

Since the identification of activating mutations of BRAF in melanoma, the technology for detection has improved dramatically. Standard mutational testing for BRAF in tumor tissue typically utilizes techniques such as bidirectional direct fluorescent sequencing and allele-specific polymerase chain reaction which are commercially available and offer high specificity. The sensitivity of these assays, however, is limited in that they are only able to detect the mutation if the tumor cells constitute >5–10% of the specimen submitted for genetic analysis [16, 17]. While this degree of sensitivity is typically sufficient to detect the presence of the BRAF^V600E^ mutation in a homogenous tumor nodule, this is likely not sensitive enough to detect a few tumor cells in the background of a high percentage of stromal or lymphatic elements, infiltrating lymphocytes, or peripheral blood cells. 

One concern regarding the utilization of mutation detection techniques with enhanced sensitivity is that a positive test might actually reflect the detection of a small subset of mutant cells. While this might have interesting scientific consequences, the clinical relevance of a tumor containing a small amount of mutant BRAF cells is none, as these patients would not be expected to benefit from BRAF inhibitors. This concern is warranted, as tumor heterogeneity has been described in primary melanomas [18]. In addition, while BRAF mutations are seen in the great majority of melanocytic nevi, vertical growth phase melanomas, and metastatic melanoma, they are rarely detected in radial growth phase melanomas (10%), which is thought to be the initial malignant lesion prior to a frankly invasive lesion [19]. This suggests that BRAF mutation may actually be an acquired event in early melanoma that leads to clonal expansion and tumor progression. Such polyclonality has not been seen in individual metastatic tumors nor when tumors across multiple sites from individual patients are sampled [18, 20]. Nevertheless, the application of enhanced sensitivity mutational analysis may not be just testing tumor samples but detecting small numbers of representative tumor cells in a background of nonmalignant cells such as in lymph nodes and peripheral blood.

More advanced techniques and assays have been developed which either provide increased sensitivity or obviate the need for increased sensitivity. These next generation tests allow for more accurate testing on samples which contain only a small amount of tumor, as well as for the detection mutations in various peripheral blood components (i.e., lymphocytes, mononuclear cells, plasma, serum). The utility of many of these tests have been explored in samples from melanoma patients with varying results.

Amplification refractory mutation systems (ARMSs) are a recently described, allele-specific technique which has enhanced sensitivity (able to detect mutation sample containing 1% mutant cells) compared to standard DNA sequencing of formalin fixed paraffin-embedded (FFPE) tissues [21]. Another approach which greatly enhances sensitivity for mutation detection is the utilization of assays which selectively amplify mutant DNA/RNA in a sample. Using a combination of allele-specific primers and locked nucleic acid primers, the detection of 10 melanoma cells in 1 mL of blood has been described [22]. A third approach to increase the sensitivity of mutation detection is reported to be able to detect one mutant cell in a thousand nonmutant cells, taking advantage of a unique restriction enzyme site in the wild-type alleles which allows for the digestion of the wild-type alleles and thus the enrichment of the mutant alleles [23]. Finally, the incorporation of COLD-PCR leads to a near doubling of sensitivity in the detection of BRAF mutation from FFPE tissue when using standard sequencing and pyrosequencing [24]. 

In addition to new technologies (ARMS) and modifications to routine techniques which lead to a greater sensitivity of mutation detection, the application of standard assays on previously untested samples is also changing how we approach BRAF testing. BRAF analysis on free DNA in the serum and plasma has been reported as has the detection of BRAF mutations from isolated, circulating tumor cells (CTCs) [25–30]. While CTC, serum and plasma BRAF analysis appears possible, it is yet to be determined whether there will be routine clinical use for one or more of these assays or if this will remain as only an experimental approach. 

While the role of standard and experimental molecular diagnostics is being utilized to identify specific mutations of interest (i.e., BRAF^V600E^), both in tissue or blood, it also may be worthwhile to test for other mutations and anomalies as these may indicate sensitivity to a particular treatment. For example, Sequenom MassARRAY technology is being used to query larger panels of oncogenic mutations, using a primer extension reaction followed by mass spectrometry to detect the products and identify mutations with potential clinical consequences [31, 32]. Array comparative genome hybridization (aCGH) offers the opportunity to examine the entire genome for copy number changes, including both amplifications and deletions that may confer sensitivity to a targeted therapy [33]. However, all these technologies are obviously limited in that they can only identify known, preselected anomalies. Whole genome analysis (WGA) has the potential to not only consolidate all or most of these modalities and tests to a single technology platform but also to identify additional genetic changes outside the design parameters of these other assays [34]. WGA also offers the opportunity to uncover previously unknown (perhaps patient-specific) mutations in melanoma genomes and to explore whether particular profiles of mutations or polymorphisms may be predictive of benefit from a particular therapy (i.e., BRAF inhibitors, HD IL-2) [35]. Still, the clinical utility of these “Next Generation” tests in the care of patients with melanoma is completely unknown.

## 4. Inhibitors of RAF Signaling (Less to More Specific Mutant BRAF, CRAF, MEK, Perhaps Mention ERK Inhibitors)

A series of small molecule inhibitors have been developed which target, with varying selectivity, wild-type BRAF, BRAF^V600E^, other mutant BRAF (at the 600 and 601 position), and CRAF. In addition, inhibitors of the downstream mediators of RAF activation, namely MEK and ERK, are also being developed. In this section, only agents which have been tested clinically and reported publically are reviewed.

## 5. BRAF Inhibitors

### 5.1. Sorafenib

Sorafenib, a multitargeted tyrosine kinase inhibitor of BRAF, CRAF, platelet-derived growth factor receptor (PDGFR), vascular endothelial growth factor receptor (VEGFR) 2, p38, and CKIT which was the first RAF-inhibitor actively studied in patients with melanoma as it was available for phase II testing in the same year in which BRAF mutations were first reported. Unfortunately, despite being evaluated in numerous phase I, II, and III studies as a single agent and in combination with chemotherapy, the clinical utility of sorafenib has been disappointing. For example, in a single agent trial of sorafenib, the median progression-free survival for patients with melanoma was 11 weeks [36]. Six patients (16%) had stable disease at 6 months that persisted for more than 12 months in some cases. However, only one of the 37 patients in the study had an actual response evaluation criteria in solid tumor (RECIST-) defined tumor response. 

This study was followed by several trials of sorafenib in combination with various cytotoxic agents, though the combination which was best studied was sorafenib, carboplatin, and paclitaxel [37–42]. Initial promise with this regimen was described in a phase I trial of sorafenib in combination with carboplatin and paclitaxel in patients with solid tumors, where 24 patients with advanced melanoma were enrolled [39]. Ten patients with melanoma (42%) achieved an objective response, and an additional 11 patients (46%) had stable disease based on RECIST. The median progression-free survival was 43.7 weeks. These promising results led to a phase III trial comparing carboplatin/paclitaxel ± sorafenib in patients with melanoma that had progressed following temozolomide or DTIC therapy. This trial (the PRISM study) enrolled 270 patients and showed no benefit for the addition of sorafenib to carboplatin/paclitaxel in this second-line patient population [40]. The combination of carboplatin/paclitaxel and sorafenib was also compared to carboplatin/paclitaxel in a treatment naïve population of patients with advanced melanoma in a placebo-controlled randomized phase III trial performed within the United States Intergroup (E2603). This trial enrolled 800 patients and found no benefit for the addition of sorafenib on either median PFS or OS [41].

### 5.2. Higher Potency BRAF Inhibitors (PLX4032, GSK2118436)

One major explanation proposed for the ineffectiveness of sorafenib as a single agent in patients with melanoma is its inability to completely inhibit BRAF, and in particular, BRAF containing the V600E mutation. Other inhibitors of BRAF, such as PLX-4032 and GSK2118436, have been developed and are more potent and selective inhibitors of mutant BRAF than sorafenib [6, 7]. This enhanced inhibition of BRAF^V600E^ predictably has led to improved clinical activity of these agents compared to sorafenib. 

### 5.3. Vemurafenib

Vemurafenib was the first higher potency BRAF inhibitor to complete phase I testing and show significant clinical benefit [42]. In the phase I trial of PLX4032, 11 of the 16 patients with tumors bearing the BRAF^V600E^ mutation who received a dose ≥240 mg twice daily in the dose escalation phase experienced tumor responses while no clinical responses were seen in the five patients with wild-type BRAF-containing tumor. In addition, 26 of 32 (81%) patients with the BRAF^V600E^ mutant melanomas treated in an expansion cohort at the recommended phase II dose of 960 mg twice daily had a clinical response, including two patients achieving a complete response (CR). The estimated median PFS was seven months which compares favorably to previously available therapies for metastatic melanoma. Further, treatment with vemurafenib leads to a reduction in levels of phosphorylated ERK (pERK) in tumors containing the BRAF^V600E^ mutation which is associated with clinical response [43, 44]. Likely, this inhibition of pERK enhances the splicing of the proapoptotic BCL-2 family member BIM thereby promoting apoptosis of BRAF^V600E^ cells [45]. 

These findings quickly led to the rapid accrual of both a single-agent phase II study (BRIM2) and a randomized controlled phase III trial (BRIM3). The phase II trial enrolled 132 patients with advanced melanoma who had received one prior therapy. The objective response rate (ORR) was 53% with a CR rate of 5%, and the progression-free survival was 6.7 months [46]. In the phase III trial, 675 patients with advanced melanoma were randomized, to either the vemurafenib or dacarbazine as front-line therapy [47]. At the first interim analysis, treatment with vemurafenib was associated with a significant reduction in the risk of death and the risk of death (63% reduction) or disease progression (74% reduction), as well as a much higher ORR (48% versus 5%). These findings served as the basis for the FDA approval of vemurafenib in August 2011. 

### 5.4. GSK2118436

GSK2118436 is a second higher potency BRAF inhibitor which has shown substantial clinical activity. In a phase I/II trial, similar to PLX4032, patients with the BRAF^V600E^ mutation treated at the two highest dose levels (150 mg twice daily and 200 mg twice daily) had a high response rate (10/16 patients, 63%) [48]. In the eight patients with non-BRAF^V600E^ mutations (V600K, V600G, and K601E) treated at a dose of ≥100 mg twice daily, three had a partial response. Both of the patients with BRAF^K601E^ progressed after first restaging, suggesting that only patients with BRAF mutations at the 600 position will respond to therapy.

## 6. MEK Inhibitors

Inhibitors of MEK, the downstream mediator of RAF activation, and the only known substrate of BRAF have shown promise in preclinical studies in melanoma and have begun to be investigated in the clinic with some encouraging results. MEK inhibitors may also be most useful in patients with BRAF^V600E^ mutation; as mutational status correlates strongly with response to MEK inhibition in murine melanoma xenograft models [49].

### 6.1. AZD6244

Two phase I trials of AZD6244 involving patients with advanced solid tumors showed this agent to be well tolerated and to possess some antitumor activity in patients with melanoma [50, 51]. In the first trial, three of eight with advanced melanoma patients treated with AZD6244 achieved a partial response; BRAF and NRAS mutational status was unavailable [50]. While in the second phase I study, only one response was seen in fourteen patients with melanoma, though this subject had a documented BRAF mutation and a complete response ongoing for over two years at the time of publication [51]. 

In addition, AZD6244 has shown promising results in murine models, particularly in combination with chemotherapy, setting the stage for combination trials [52]. Building upon this, a pilot study of AZD6244 in combination dacarbazine, docetaxel, or temsirolimus in patients with advanced melanoma was performed [53]. Eighteen patients were treated in whom BRAF and NRAS mutational status was known. Clinical response was seen in five out of nine patients (55%) with a BRAF mutation, whereas no responses were seen in any of the nine patients without a BRAF mutation which included four patients with an NRAS mutation. In addition, time to progression was significantly improved in patients with a BRAF mutation compared to those patients without (median 31 weeks versus 8 weeks).

### 6.2. GSK1120212

GSK1120212 is a reversible, selective inhibitor of MEK1/MEK2 which has been shown, in a phase I trial, to have single-agent efficacy in patients with advanced, BRAF^V600E^ mutant melanoma [54]. Specifically, eight of 20 patients with BRAF mutant melanoma treated with GSK2110212 had a confirmed response with two patients achieving a CR. Interestingly, two of 22 patients with wild-type BRAF had a PR with treatment, suggesting that some melanoma tumors are dependent on ERK/MAP kinase signaling despite the absence of a BRAF mutation.

### 6.3. PD-0325901

A phase I trial of PD-0325901 enrolled 48 patients with advanced melanoma, of whom 3 (6%) had a confirmed PR, 10 (21%) had stable disease for ≥4 months, and a total of 15 (31%) patients showed reduction in Ki-67 tumor staining [55]. Mutational analysis data of these patients was not provided.

### 6.4. AS703026

Similar results have been recently reported with AS703026, which is a potent MEK1/2 inhibitor. In the phase I study, three of eight patients had a partial response with treatment in one of two treatment schedules [56]. Mutational status of the melanoma patients was not reported.

While the clinical data on MEK inhibitors is encouraging, it is quite preliminary. The true value of these agents must await phase II and phase III trials in patients with BRAF mutant melanoma. One such trial that is currently ongoing is a randomized, phase III trial of GSK1220212 compared with chemotherapy (either dacarbazine or paclitaxel) in patients with melanoma harboring BRAF mutations (NCIT01245062). 

## 7. Emerging Mechanisms of Resistance to BRAF Inhibition

Importantly, it appears that the great majority of patients treated with single agent PLX-4032 will eventually exhibit disease progression despite successful inhibition of the BRAF^V600E^ and a high rate of objective response early in the course of therapy. Preliminary studies suggest that resistance to PLX-4032 is not related to the development of a second mutation which impairs the binding of the treatment drug to BRAF, a resistance mechanism noted for targeted therapy in other malignancies such as nonsmall cell lung cancer, chronic myelogenous leukemia, and gastrointestinal stromal tumor [57–59]. Instead, resistance is mediated by reactivation of the MAPK pathway in most tumors through alternative means.

It is from in vitro studies of BRAF^V600E^-mutated cells which have been generated to exhibit acquired resistance to BRAF inhibitors, that has led to the first clues as to how BRAF-mutated cells are able to survive BRAF inhibition. It appears clear that reestablishment of MAPK signaling is the key variable in acquired resistance to BRAF inhibition [60–63]. This can be achieved through upregulation of receptor tyrosine kinases (i.e., PDGFRB, ERBB2) [61, 62], activation of RAS [60, 62], upregulation of CRAF [61, 63], activation of the Ser/Thr MAPK kinases (COT) [61], and development of a secondary activating mutation in MEK [64, 65]. In addition, signaling through the PI3K pathway initiated by insulin growth factor receptor 1 (IGF-1R) is an alternative mechanism of acquired resistance which has also been described [66]. Of note, each of these mechanisms have been investigated and corroborated in small numbers of tumor samples from patients who had biopsies performed at the time of resistance, and dependence on those upregulated or mutated signaling mediators was not demonstrated. 

Primary resistance to BRAF inhibition is seen in less than 10% of patients with BRAF mutant melanoma treated with vemurafenib [42]. While there is no data from clinical samples which helps to identify which patients are likely not to benefit from BRAF inhibitors, preclinical studies suggest that elevated pretreatment levels of CRAF, as well as, baseline CCND1 amplification in tumors, leading to downstream overexpression of Cyclin D1 and enhanced CDK4 expression, are promising pretreatment biomarkers worth further investigation [63, 67]. 

In BRAF wild-type (BRAF^WT^) melanoma cells, the MAPK kinase pathway is activated by vemurafenib (and the analogous PLX4720) leading to upregulation of MEK and ERK and enhanced proliferation [68, 69]. This appears to be secondary to the activation of CRAF with subsequent downstream signaling through MEK and ERK with expected oncogenic consequences in BRAF^WT^ cells [69]. Further, this CRAF activation appears to be mediated through the formation of a heterodimer with the BRAF^WT^ protein and/or CRAF homodimer which is most apparent in RAS-mutated cells [70, 71]. In addition, PLX4720 enhances the levels of the antiapoptotic BCL-2 family member protein MCL-1 in NRAS mutant melanoma cells through enhanced signaling through the MAPK pathway [72]. While it is clear that CRAF activation and enhanced MAPK pathway signaling occur in BRAF^WT^ melanoma cells (particularly those which harbor an NRAS mutation) treated with BRAF inhibitors such as vemurafenib and PLX4720, the clinical relevance of this is uncertain. Specifically, it is not thought that clinically acquired resistance to vemurafenib, for example, occurs solely due to growth of a subset of BRAF^WT^ melanoma cells as persistence of the BRAF^V600E^ mutation has been identified in all tumors analyzed and reported to date. In fact, it appears that specific changes in BRAF^V600E^-mutated cells allow for adaptations which lead to renewed growth despite continued BRAF inhibition.

## 8. Future Directions

The establishment of single-agent efficacy of selective BRAF inhibitors, and to lesser extent MEK inhibitors, is a major breakthrough for the treatment of patients with BRAF mutation positive melanoma. Though most patients treated with these agents are predicted to progress on treatment, the elucidation of the mechanisms of resistance described above helps guide future sequential and combinational therapy. Based on the finding that MAPK pathway activity is reactivated in melanoma following selective BRAF inhibition, the first combination trial of a selective BRAF inhibitor (GSK2118436) with a MEK inhibitor (GSK1120212) is underway and appears tolerable with both agents being administered at their standard single-agent doses [73]. In addition to this combination, trials with selective BRAF inhibitors either in combination with or followed by IGF-R1 antagonists and other receptor tyrosine kinase inhibitors might be anticipated from the results of preclinical models of resistance [61, 62, 66]. 

Another approach to enhancing the effectiveness of selective BRAF inhibitors and MEK inhibitors is the addition of agents which might augment apoptosis. One such agent is ABT-263 which is a BH3-mimetic currently in clinical development. In preclinical studies, the less bioavailable homologous BH3-mimetic ABT-737 in combination with a MEK inhibitor led to enhanced lethality compared to either agent alone [74]. Whether ABT-263 in combination with MEK or selective BRAF inhibitors will improve clinical outcomes is unknown, though it is perhaps worth exploring in an early-phase clinical trial.

In addition to combination therapy with molecularly targeted agents which inhibit signaling induced by selective BRAF inhibition or promote apoptosis, another promising approach to maximizing the benefit of BRAF or MEK inhibiters is to combine these agents with immunotherapy. Recently, immune checkpoint inhibitors, including the anti-CTLA-4 monoclonal antibody ipilimumab and the anti-PD monoclonal antibody MDX-1106, have shown single agent efficacy in patients with metastatic melanoma [75, 76]. Importantly, it appears that PLX4032 does not adversely affect human T-lymphocytes (T-cells) function while MEK inhibitors do [77]. Further, vemurafenib has been shown to improve immune recognition by antigen-specific T cells in melanoma [78]. These findings provide a rationale for a study evaluating the safety and efficacy of selective BRAF inhibition in combination with immunotherapy, including ipilimumab, MDX1106, and possibly high-dose IL2.

## 9. Conclusions

It had been hoped for many years that the growing understanding of the molecular pathways involved in melanoma development and the increasing availability of specific inhibitors of these pathways would enable the rational development of future therapies. With the emergence of vemurafenib and GSK2118436, the first molecularly targeted agents to lead to tumor responses in a large percentage of patients, a new approach to the treatment of melanoma has begun. As a result, all patients with advanced melanoma should have BRAF mutational analysis prior to commencing systemic therapy. In those patients whose tumors harbor a mutation in BRAF, every attempt should be made to treat these patients with either of the two highly potent BRAF inhibitors. Additionally, as more is learned about the resistance mechanisms to BRAF inhibitors, the development of combination trials of novel molecularly targeted therapies can be expected; further clinical improvements can only be sorted out by carefully conducted preclinical and clinical studies that include pretreatment and on-treatment biopsies. With BRAF being established as the first point of vulnerability in melanoma, it is hoped that a molecular understanding of the limits of BRAF inhibition will lead to further clinical benefit.
